# Material Degradation Inverse Identification for Cantilever Beams Using Experimental Frequency Response Function

**DOI:** 10.3390/s26041266

**Published:** 2026-02-15

**Authors:** Qi Chen, Carol Featherston, David Kennedy, Abhishek Kundu

**Affiliations:** School of Engineering, Cardiff University, Cardiff CF24 3AA, UK

**Keywords:** Hamiltonian Monte Carlo, Bayesian inference, Karhunen-Loève expansion, experimental FRF, physical constraint enforcement, two-phase regularization, stiffness regularization, structural damage identification

## Abstract

This paper presents a stochastic framework for the inverse identification of structural material degradation (SMD) in cantilever beams. The method combines the Karhunen–Loéve (KL) expansion for the efficient parameterisation of spatially varying material decay with experimental Frequency Response Function (FRF) data within a Bayesian inference scheme. This approach employs a low-dimensional spectral parameterisation via the KL expansion, which mitigates the curse of dimensionality inherent in element-wise model updating, and provides a full-field probabilistic description of SMD. A two-phase constraint strategy was developed to address the fundamental tension between physical plausibility and algorithmic stability of the inverse identification algorithm: (1) physical regularisation during identification stabilises the ill-posed inverse problem, and (2) post-convergence selective regularisation eliminates physically impossible stiffness enhancements (exceeding 1.1 × baseline) that arise from measurement and modelling uncertainties. This phased approach prevents the algorithm distortion that occurs when constraints are applied too stringently during iteration, while ensuring final results respect fundamental physical principles. The framework is experimentally validated on a steel cantilever beam with a symmetric open-edge cut. Laser vibrometry measurements under swept-sine excitation demonstrate successful localisation and quantification of SMD, with the 95% credible interval accurately capturing the damaged region after physical constraint application. The adaptive constraint strategy resolves the delicate balance between mathematical stability and physical plausibility in inverse identification.

## 1. Introduction

Structural health monitoring (SHM) has become essential for ensuring the safety, reliability, and serviceability of engineering structures throughout their operational life. Beams—widely used in bridges, buildings, and mechanical systems—are especially critical as their damage can significantly compromise overall structural performance. Common forms of damage, such as cracking, corrosion or material degradation, can lead to local stiffness reduction, altered vibration characteristics, and ultimately performance loss or catastrophic failure [1,2,3]. Therefore, accurate and timely identification of beam damage is crucial for preventive maintenance and life-cycle management.

Inverse analysis has emerged as a powerful tool for structural diagnosis, allowing the estimation of internal damage parameters from measurable system responses (as surveyed in the Handbook of Damage Mechanics; [4]). Vibration-response-based inverse identification methods have attracted considerable attention in recent decades because they offer non-destructive and globally sensitive means to detect structural damage. Among these, the Frequency Response Function (FRF) analysis is particularly effective, capturing the dynamic relationship between input excitation and structural response in the frequency domain [5]. As highlighted in a recent state-of-the-art review, FRF-based techniques continue to be a vibrant area of research due to their rich information content, with ongoing developments aimed at improving their accuracy and computational efficiency for damage identification [6]. Furthermore, contemporary studies are extending FRF concepts to nonlinear system behaviours for enhanced damage sensitivity [7]. Changes in FRF characteristics—such as resonant frequency shifts, damping variations, or amplitude/phase distortions—can indicate stiffness loss or mass redistribution caused by damage [8,9]. Experimental FRFs provide an empirical basis for damage identification since they reflect actual boundary conditions and uncertainties inherent in physical systems. While FRF-based approaches offer rich dynamic information, alternative methodologies such as strain modes-based interval damage identification have demonstrated enhanced sensitivity to local stiffness changes [10]. However, these methods often rely on extensive sensor networks and may be less suited for full-field degradation identification where spatial variability is significant.

Interpreting FRF variations becomes challenging when the material properties of a structure are spatially heterogeneous or gradually degrade over time. In practice, such variability is unavoidable due to manufacturing imperfections, environmental exposure, or fatigue-induced microstructural changes [3]. To represent these spatial uncertainties in a physically consistent manner, stochastic field modelling has been introduced into structural analysis. The Karhunen–Loève (KL) expansion offers a mathematically rigorous and computationally efficient framework for representing random fields with reduced dimensionality [11,12]. By expressing a stochastic property (e.g., Young’s modulus) as a series of orthogonal eigenfunctions weighted by uncorrelated random variables, the KL expansion can accurately simulate spatial variability and degradation in material stiffness [13,14,15].

Integrating the KL-based stochastic representation with experimental FRF measurements provides a powerful framework for probabilistic damage identification. Simulated degradation scenarios generated via the KL expansion can be compared with experimental FRF data to infer the most probable damage patterns, enabling robust detection and quantification of material deterioration under uncertainty [16,17]. This combined approach enhances both the interpretability and reliability of vibration-based SHM systems.

Nevertheless, inverse identification of material degradation from FRF measurements constitutes an inherently ill-posed problem. Multiple stiffness distributions can produce similar dynamic responses, and measurement noise can lead to solution instabilities. Bayesian inference [18,19] with Hamiltonian Monte Carlo (HMC) sampling provides a principled framework for uncertainty quantification, but practical implementation reveals a critical tension between mathematical regularisation (required for stability) and enforcement of physical constraints (essential for plausibility). Overly stringent regularisation during iterative sampling can distort convergence, while insufficient constraints yield physically impossible results such as stiffness enhancements in damaged regions. This methodological gap limits the practical application of stochastic identification frameworks.

To address these challenges, this paper presents a novel stochastic framework with three key contributions. First, we combine KL expansion for efficient degradation parameterisation with experimental FRF data within a Bayesian inference scheme, circumventing the “curse of dimensionality” associated with element-wise approaches while providing a probabilistic description of structural material degradation (SMD). Second, we introduce a two-phase constraint strategy that separates mathematical stabilisation during HMC sampling from post-convergence physical bound enforcement. This prevents algorithmic distortion while ensuring final results respect fundamental physical principles. Third, we experimentally validate the framework on a steel cantilever beam with symmetric open-edge cuts, demonstrating successful damage localisation and quantification using laser vibrometry measurements.

The present study therefore focuses on beam damage identification using experimental FRFs in conjunction with KL expansion-based modelling of material degradation. The goal is to investigate how spatial variability in stiffness affects the dynamic response, and how FRF-based indicators can be employed to identify and quantify degradation while addressing the regularisation-plausibility trade-off. Recent advances in structural identification have increasingly adopted hybrid physics-data-driven frameworks to enhance robustness and dimensionality reduction for ill-posed inverse problems under uncertainty. For instance, ref. [20] developed a reduced-order modelling approach combining principal component analysis (PCA) with a physics-data-driven neural network (PDNN) for aerodynamic load inversion under interval field uncertainties, demonstrating effective integration of physical constraints with data-driven mappings in high-dimensional spatial identification tasks. In a similar spirit, the present study employs KL expansion to represent spatially-varying stiffness degradation, but focuses specifically on experimental FRF-based Bayesian identification and introduces a novel two-phase constraint strategy to explicitly decouple mathematical regularisation from post-convergence physical plausibility enforcement—a methodological distinction from prior hybrid frameworks. A cantilever beam case study underscores the method’s accuracy, robustness, and potential for deployment in aerospace, civil, and mechanical engineering applications. The outcomes are expected to contribute to a probabilistic, data-informed framework for evaluating structural health in beam-like systems.

A key methodological contribution of this work is the development of a two-phase constraint strategy that explicitly decouples the numerical stabilisation required during HMC sampling from the post-convergence enforcement of physical plausibility. While regularisation is commonly employed in ill-posed inverse problems—e.g., through Tikhonov regularisation, Bayesian priors, or ad hoc filtering—existing single-phase approaches often face a fundamental conflict: overly strict constraints applied during iterative optimisation can distort convergence, trap sampling in local minima, or prevent the exploration of parameter space necessary for accurate damage identification. Recent adaptive regularisation methods, such as the Adaptive Probabilistic Regularisation (APR) approach that applies parameter-wise weighting during iteration [21], have improved convergence efficiency but still enforce physical bounds within the iterative loop. In contrast, insufficient regularisation leads to physically implausible results, such as non-physical stiffness enhancements in damaged regions. The proposed two-phase strategy resolves this tension by applying moderate, mathematically motivated regularisation during inference to stabilise the ill-posed problem, followed by selective, element-wise physical bound enforcement after convergence. This ensures that the final stiffness field respects fundamental physical principles—namely, that damage reduces, rather than increases, structural rigidity—without compromising the algorithm’s ability to locate and quantify degradation during the sampling phase. To our knowledge, this explicit separation of mathematical stabilisation and physical plausibility enforcement represents a novel contribution to the Bayesian inverse identification of structural damage, particularly in the context of FRF-based updating under experimental uncertainty.

The remainder of this paper is organised as follows. Section 2 presents the spectral parameterisation of structural systems using KL expansion. Section 3 develops the Bayesian inverse framework with the proposed two-phase constraint strategy. Section 4 details the experimental methodology and apparatus selection. Section 5 presents identification results and discusses the efficacy of the constraint approach. Finally, Section 6 concludes with implications for practical SHM applications.

## 2. Spectral Parameterisation of Structural Dynamic Systems

### 2.1. Mechanical Framework for Structural Material Degradation

SMD in beam-like structures fundamentally manifests as a reduction in effective bending stiffness EI(x) along the beam span. This stiffness reduction originates from various physical mechanisms that alter the material’s constitutive behaviour at the micro- or meso-scale:

- Localised damage: Cracks, notches, or cut-outs that create stress concentrations and reduce effective cross-sectional area. In our experimental validation, symmetric open-edge cuts represent this category.
- Distributed degradation: Corrosion, wear, or fatigue-induced micro-cracking that gradually reduces material integrity over larger regions.
- Material property evolution: Changes in Young’s modulus *E* due to environmental exposure (moisture, temperature), chemical changes, or phase transformations.
- Geometric alterations: Thickness reduction or cross-sectional area loss without changes to intrinsic material properties.

For an Euler–Bernoulli beam, the bending stiffness depends on both material and geometric properties. As an illustration, for a rectangular cross-section beam, it takes the form:

(1) $$EI\left(x\right)=E\left(x\right)\cdot I\left(x\right)=E\left(x\right)\cdot \frac{b\cdot h{\left(x\right)}^{3}}{12}$$

where E(x) is the spatially-varying Young’s modulus, *b* is the constant width, and h(x) is the potentially varying thickness. In our framework, we model the combined effect through an effective stiffness EI(x), acknowledging that the inverse problem cannot uniquely separate material (*E*) from geometric (*I*) contributions without additional measurements.

We adopt a lumped parameterisation where stiffness reduction is represented through the KL expansion (as discussed in (11)):

(2) $$EI\left(x,\xi \right)=\bar{EI}\cdot \left(1+\sum_{n=1}^{M}\xi_{n}\sqrt{\lambda_{n}}f_{n}\left(x\right)\right)$$

where $\left\{\lambda_{n}\right\}$ and $\left\{f_{n}\left(x\right)\right\}$ are the eigenvalues and orthonormal eigenfunctions, respectively, the $\bar{EI}$ represents the mean function of the random stiffness field and *M* is the truncation order of the KL expansion. This approach captures both localised damage and distributed degradation within a unified stochastic framework.

### 2.2. Karhunen–Loève Representation of the Material Property Field

In reality, material properties such as Young’s modulus are not deterministic but exhibit random spatial fluctuations. A deterministic field is insufficient to capture this variability or the associated uncertainty in model predictions. To address this, we adopt a probabilistic representation using the theory of random fields. Following the stochastic modelling framework in [22], the spatially varying material property is represented by a second-order random field

(3) $$W\left(x,\theta \right),\;x\in D\subset R^{d},$$

where *D* denotes the spatial domain of the structure (d=1,2,3 for one-, two-, and three-dimensional problems, respectively), and θ denotes the sample space coordinate in the underlying probability space. The field has a mean function

$$\bar{W}\left(x\right)=E\left[W\left(x,\theta \right)\right]$$

and covariance kernel

(4) $$C\left(x_{1},x_{2}\right)=\mathrm{Cov}\;\left(W\left(x_{1},\theta \right),W\left(x_{2},\theta \right)\right).$$

The covariance function is known to be bounded, symmetric, and positive semi-definite [23,24,25]. Therefore, the corresponding covariance operator is compact and self-adjoint, and the kernel admits the classical spectral decomposition [26]

(5) $$C\left(x_{1},x_{2}\right)=\sum_{n=1}^{\infty }\lambda_{n}f_{n}\left(x_{1}\right)\;f_{n}\left(x_{2}\right),$$

where $\left\{\lambda_{n}\right\}$ and $\left\{f_{n}\left(x\right)\right\}$ are the eigenvalues and orthonormal eigenfunctions, respectively.

The field is decomposed as

(6) $$W\left(x,\theta \right)=\bar{W}\left(x\right)+\beta \left(x,\theta \right),$$

where β is the zero-mean fluctuation part. Since β retains the covariance of *W*, application of the KL expansion yields

(7) $$\beta \left(x,\theta \right)=\sum_{n=1}^{\infty }\xi_{n}\left(\theta \right)\sqrt{\lambda_{n}}\;f_{n}\left(x\right),$$

where the coefficients are obtained by orthogonal projection,

(8) $$\xi_{n}\left(\theta \right)=\frac{1}{\sqrt{\lambda_{n}}}\int_{D}\beta \left(x,\theta \right)\;f_{n}\left(x\right)\;dx,$$

and satisfy

$$E\left[\xi_{n}\right]=0,\;E\left[\xi_{m}\xi_{n}\right]=\delta_{mn}.$$

In many structural materials, the fluctuation field arises from the accumulation of numerous weak microstructural variations. Under this widely accepted small-fluctuation assumption, the central-limit tendency motivates modelling β(x,θ) as approximately Gaussian. Because the KL coefficients in Equation (8) are linear functionals of β, they can therefore be treated as independent Gaussian variables,

(9) $$\xi_{n}\left(\theta \right)∼N\left(0,1\right),$$

consistent with common stochastic finite element modelling practice.

Combining the mean and fluctuation components, the field admits the KL representation

(10) $$W\left(x,\theta \right)=\bar{W}\left(x\right)+\sum_{n=1}^{\infty }\xi_{n}\left(\theta \right)\sqrt{\lambda_{n}}\;f_{n}\left(x\right).$$

For computational purposes, the series is truncated to the first *M* modes:

(11) $$W_{M}\left(x,\theta \right)=\bar{W}\left(x\right)+\sum_{n=1}^{M}\xi_{n}\left(\theta \right)\sqrt{\lambda_{n}}\;f_{n}\left(x\right).$$

The KL expansion’s efficacy for representing spatially correlated engineering uncertainties, such as material degradation, continues to be demonstrated in recent probabilistic structural analyses [27]. Given that material covariance kernels typically exhibit rapidly decaying eigenvalues, only a modest number of modes is required for an accurate and efficient representation.

The truncation error, expressed as the unresolved variance, is

(12) $$E\;\left[∥W-W_{M}{∥}_{L^{2}\left(D\right)}^{2}\right]=\sum_{n=M+1}^{\infty }\lambda_{n}.$$

Thus, the retained modes capture the proportion

(13) $$\eta_{M}=\frac{\sum_{n=1}^{M}\lambda_{n}}{\sum_{n=1}^{\infty }\lambda_{n}},$$

of the total variance.

In practice, *M* is chosen so that $\eta_{M}$ lies between 90% and 99%, ensuring that the residual variance is negligible for engineering use. Given that material covariance kernels typically exhibit rapidly decaying eigenvalues, only a modest number of modes is required for an accurate and efficient representation.

### 2.3. Stochastic Representation of System Matrices and Frequency-Domain Response

The structural dynamic system is governed by the second-order matrix differential equation:

(14) $$M\ddot{u}\left(t\right)+C\dot{u}\left(t\right)+Ku\left(t\right)=F\left(t\right),$$

where M, C, and K denote the mass, damping, and stiffness matrices, respectively, and F(t) is the applied force vector. In practical applications, these matrices may exhibit variability due to uncertainties in material properties (e.g., Young’s modulus *E*, density ρ, or damping coefficients *c*), geometric imperfections, or manufacturing inconsistencies.

Using the KL expansion for the underlying material property fields, the system matrices can be expressed as:

(15) $$\begin{array}{cc} M(\xi ) & =\bar{M}+\sum_{n=1}^{M_{M}}\xi_{n}^{M}\left(\theta \right)M_{n}, \end{array}$$

(16) $$\begin{array}{cc} K(\xi ) & =\bar{K}+\sum_{n=1}^{M_{K}}\xi_{n}^{K}\left(\theta \right)K_{n}, \end{array}$$

(17) $$\begin{array}{cc} C(\xi ) & =\bar{C}+\sum_{n=1}^{M_{C}}\xi_{n}^{C}\left(\theta \right)C_{n}. \end{array}$$

where $\bar{M},\bar{K},\bar{C}$ are the deterministic baseline matrices, $M_{n},K_{n},C_{n}$ are the deterministic component matrices arising from the KL expansion of the respective matrices, and $\xi_{n}^{M},\xi_{n}^{K},\xi_{n}^{C}$ are independent standard random variables.

These deterministic component matrices capture effects such as material loss, softening, or local changes in geometry. For example, a loss of material in a beam segment simultaneously reduces mass per unit length and bending stiffness, leading to correlated variations in M and K.

The stochastic deviations propagate from the material level to the system matrices through standard finite element formulations. For the stiffness matrix,

(18) $$K=\int_{\Omega }B^{T}D\left(\xi \right)B\;d\Omega ,$$

where B is the strain-displacement matrix and D(ξ) is the stochastic elasticity (material stiffness) matrix. For the specific case of an Euler–Bernoulli beam, the general constitutive matrix D(ξ) appearing in the finite element formulation reduces to the scalar bending stiffness EI(x,ξ) defined in Equation (2).

Similar formulations apply for M and C. This formulation is general and can accommodate both independent and coupled stochastic variations.

In the frequency domain, the equation of motion for a structural system is expressed as:

(19) Z(ω,ξ)U(ω,ξ)=F(ω),

where

(20) $$Z\left(\omega ,\xi \right)=K\left(\xi \right)+j\omega C\left(\xi \right)-\omega^{2}M\left(\xi \right)$$

is the stochastic dynamic stiffness matrix, and U(ω,ξ) is the displacement vector at all degrees of freedom.

The FRF at sensor locations is defined as:

(21) H(ω,ξ)=RU(ω,ξ),

where R is a Boolean or selection matrix that extracts only the degrees of freedom corresponding to sensor locations.

Due to the high dimensionality of Z, we do not compute its full inverse. Instead, we solve the system with stochastic linear solvers. In this instance, we have used Monte Carlo sampling to express our solution in the stochastic space:

(22) $$U\left(\omega ,\xi^{\left(s\right)}\right)=Z{\left(\omega ,\xi^{\left(s\right)}\right)}^{-1}F\left(\omega \right),\;s=1,\ldots ,N_{s},$$

where $N_{s}$ is the number of Monte Carlo samples, and $\xi^{\left(s\right)}$ is the vector of stochastic variables for the *s*-th sample.

**Figure 1:**
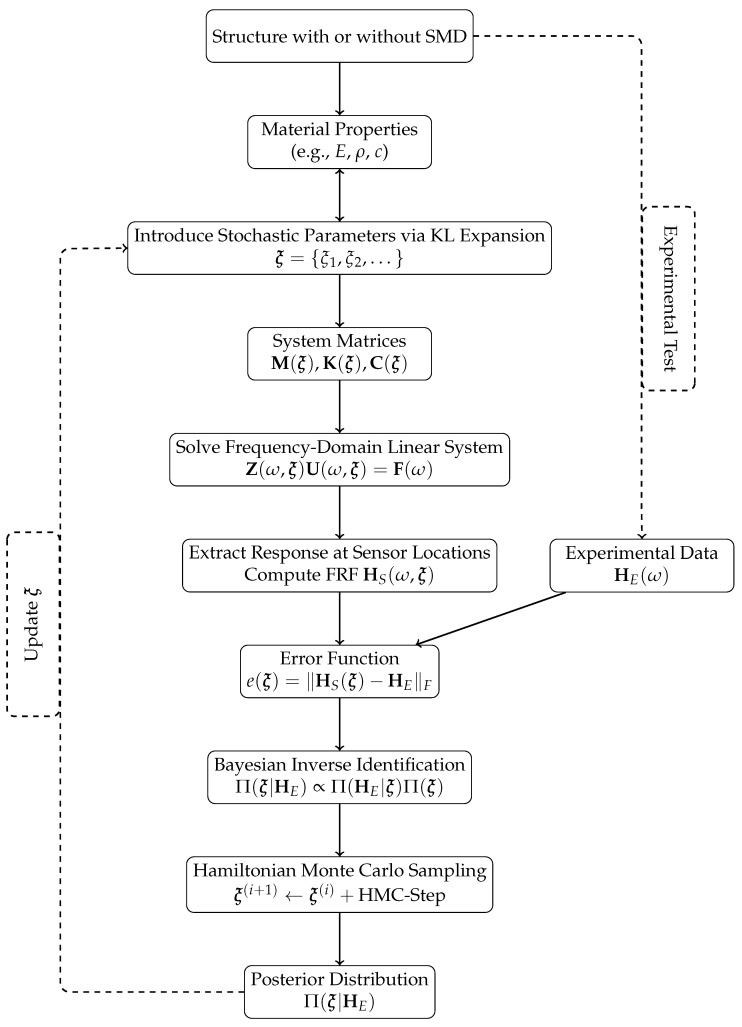
Complete stochastic identification framework: forward propagation of uncertainties and Bayesian inverse identification using HMC sampling to estimate material parameters from experimental data.

This approach naturally incorporates stochastic variations in the mass, stiffness, and damping matrices via KL expansion as shown in Figure 1. The method accommodates both independent and interdependent variations, such as those arising from material degradation.

## 3. Inverse Problem Regularisation Sensor Position Selection

Identifying structural damage from FRFs is an ill-posed inverse problem complicated by measurement noise, model uncertainty, and non-unique solutions. This section presents an integrated framework to overcome these challenges. First, a Bayesian inference approach quantifies parameter uncertainty. Second, a physics-based regularisation strategy stabilises the solution by constraining the bending rigidity field. A two-phase implementation separates iterative stabilisation from post-convergence physical bound enforcement to avoid algorithmic distortion. Finally, an optimal sensor placement strategy ensures that limited measurements capture essential dynamic features. Together, these components enable robust, physically plausible damage identification under practical experimental constraints.

### 3.1. Bayesian Inverse Approach for Damage Identification

To quantify the uncertainty in material parameters given measured FRF data, this work employs a Bayesian inference framework. The core of this approach is Bayes’ theorem, which combines prior knowledge with new evidence to form a posterior distribution. For our problem, the theorem is expressed as:

(23) $$\Pi \left(\xi |H_{E}\right)\propto \Pi \left(H_{E}|\xi \right)\Pi \left(\xi \right)$$

Here, ξ is the random vector of unknown material parameters to be identified, Π(ξ) is the prior distribution representing our initial beliefs, $\Pi (H_{E}|\xi )$ is the likelihood function quantifying the probability of observing the measured data $H_{E}$ given a set of parameters, and $\Pi (\xi |H_{E})$ is the posterior distribution we seek, which represents the updated belief about the parameters after considering the evidence.

The prior distribution for the parameters ξ is chosen to be a standard multivariate Gaussian for computational tractability, as discussed in Section 2.2, Equation (9):

(24) $$\Pi \left(\xi \right)=\frac{1}{{\left(2\pi \right)}^{M/2}}\exp\left(-\frac{1}{2}\xi^{T}\xi \right)$$

The likelihood function connects the model to the physical measurement data. The accuracy of the structural model in simulating material damage is evaluated by comparing the simulated FRF, $H_{S}\left(\omega ,\xi \right)$, to the experimentally measured FRF, $H_{E}\left(\omega \right)$. The discrepancy is defined by an error function, taken as the Frobenius norm:

(25) $$e\left(\xi \right)=∥H_{S}\left(\xi \right)-H_{E}{∥}_{F}$$

Assuming the measurement errors follow a Gaussian distribution with variance $\sigma^{2}$, the likelihood function is formulated as:

(26) $$\Pi \left(H_{E}|\xi \right)\propto \exp\left(-\frac{1}{2\sigma^{2}}e{\left(\xi \right)}^{2}\right)$$

This choice of a Gaussian likelihood is common but not unique; it is selected here for its suitability in modelling measurement noise within this damage identification context.

Substituting the prior and the likelihood into Bayes’ theorem (Equation (23)) yields the posterior distribution:

(27) $$\begin{array}{cc} \Pi (\xi |H_{E}) & \propto \Pi \left(H_{E}|\xi \right)\Pi \left(\xi \right)\\ \; & \propto \frac{1}{Z}\exp\left(-\frac{1}{2\sigma^{2}}{∥H_{S}-H_{E}∥}_{F}^{2}\right)\cdot \frac{1}{{\left(2\pi \right)}^{M/2}}\exp\left(-\frac{1}{2}\xi^{T}\xi \right) \end{array}$$

Consequently, the task of identifying material degradation or loss is formulated as finding the parameters ξ that maximise this posterior probability, which is equivalent to minimising the error e(ξ) while being regularised by the prior.

### 3.2. Stabilising the Identification Through Physical Regularisation

Inverse identification using FRF data is inherently ill-posed: multiple stiffness fields may reproduce similar dynamic responses, and small perturbations in the measured data can result in disproportionately large variations in the estimated parameters. To ensure stability and physical plausibility, the Bayesian formulation in Section 3.1 is complemented by a regularisation term applied directly to the bending rigidity field.

From the posterior distribution in Equation (27), we can derive the complete expression for the negative log-posterior:

(28) $$-\ln\Pi \left(\xi |H_{E}\right)=\frac{1}{2\sigma^{2}}{∥H_{S}\left(\xi \right)-H_{M}∥}_{F}^{2}+\frac{1}{2}\;\xi^{T}\xi ,\;$$

where the constant term includes the normalisation factors from both the prior and likelihood. The maximum a posteriori (MAP) estimate corresponds to the parameter vector ξ that minimises this negative log-posterior. It is important to note that this specific quadratic form arises from our choice of Gaussian likelihood and Gaussian prior; other distributional assumptions would lead to different functional forms for the MAP objective.

To incorporate additional physical constraints, we augment this objective with a regularisation term R(ξ) that penalises non-physical solutions:

(29) $$\xi_{\mathrm{MAP}}=\arg\min_{\xi }\left[\frac{1}{2\sigma^{2}}{∥H_{S}\left(\xi \right)-H_{M}∥}_{F}^{2}+\frac{1}{2}\;\xi^{T}\xi +R\left(\xi \right)\right],\;$$

where $H_{M}$ is the measured FRF, $H_{S}\left(\xi \right)$ is the simulated FRF corresponding to the parameter vector ξ, the term $\frac{1}{2}\xi^{T}\xi$ arises from the Gaussian prior on the KL coefficients, and R(ξ) is a physical regularisation term defined below.

The regularisation term is defined as

(30) $$R\left(\xi \right)={∥L\left({\hat{\kappa }}_{S}\left(x,\xi \right)-\kappa_{M}\left(x\right)\right)∥}_{2}^{2},$$

which penalises non-physical oscillations in the reconstructed rigidity field, where $\kappa_{S}$ is the simulated bending rigidity, $\kappa_{M}$ is the assumed actual rigidity field, and *L* can be a differential operator that encodes the expected smoothness based on the beam’s mechanics. Importantly, this formulation does not force $\kappa_{S}$ to coincide with any baseline value; instead, it promotes spatial smoothness consistent with the mechanics of bending and the expected form of damage. The physical regularisation term R(ξ) is designed to penalise deviations of the simulated stiffness field from a physically plausible baseline. The operator *L* can be linear (e.g., a first-difference operator to promote smoothness) or a scaling function that amplifies discrepancies in regions where stiffness enhancement is suspected. The strength of this regularisation is implicitly balanced with the data misfit term during HMC sampling. The guiding principle was to apply just enough regularisation to prevent solution divergence and oscillatory artifacts, without over-smoothing the genuine damage signature. This was monitored empirically by observing the stability of the HMC chains and the spatial coherence of the intermediate stiffness estimates (the details of the HMC can be checked in Appendix A).

Formally, the addition of the regularisation term R(ξ) modifies the posterior distribution from Equation (27) to:

(31) $$\begin{array}{cc} \Pi_{\mathrm{reg}}\left(\xi |H_{E}\right) & \propto \Pi \left(H_{E}|\xi \right)\Pi \left(\xi \right)\exp\left(-R(\xi )\right)\\ \; & \propto \frac{1}{Z}\exp\left(-\frac{1}{2\sigma^{2}}{∥H_{S}-H_{E}∥}_{F}^{2}\right)\cdot \frac{1}{{\left(2\pi \right)}^{M/2}}\exp\left(-\frac{1}{2}\xi^{T}\xi \right)\cdot \exp\left(-R(\xi )\right). \end{array}$$

The MAP estimate in Equation (29) corresponds to finding the mode of this regularised posterior.

This physical regularisation approach provides the necessary stabilisation for the ill-posed inverse problem. However, implementation revealed a critical balance: while insufficient regularisation yields mathematically unstable results, overly stringent application of R(ξ) during the iterative identification process distorts the algorithm’s convergence, potentially creating false indications of degradation. This finding motivated the development of a complementary post-convergence strategy described in Section 3.3, which applies final physical bounds without interfering with the identification iteration.

### 3.3. Post Regularisation Based on the EI Physical Meaning

The physical regularisation in Section 3.2 stabilises the identification process. Our implementation revealed that applying a single, global bound during iteration can interfere with algorithm performance, as overly stringent constraints may cause HMC sampling to converge to distorted solutions or fail to explore the parameter space properly.

To resolve this, we implement a refined, two-phase regularisation strategy:

1. Phase 1—Iterative Stabilisation: Apply the regularisation term R(ξ) with moderate weighting during HMC sampling to guide convergence without imposing rigid physical bounds (Section 3.2).
2. Phase 2—Post-Convergence Regularisation: After the algorithm converges, regularisation is applied individually to each identified flexural stiffness (EI) profile. For each sample in the posterior distribution, any EI value exceeding the physically plausible threshold is corrected. The regularised value $\kappa_{\mathrm{reg}}\left(x\right)$ is obtained as:
  (32) $$\kappa_{\mathrm{reg}}\left(x\right)=\min\left(\kappa_{\mathrm{identified}}\left(x\right),\;\alpha \cdot \kappa_{\mathrm{base}}\right),$$
  where α=1.1 provides allowance for combined experimental and numerical uncertainties. After this element-wise regularisation, the mean and credible intervals (CI) of the posterior distribution are recalculated to produce the final statistical estimates. Throughout this work, we report uncertainty using Bayesian credible intervals rather than frequentist confidence intervals. This Bayesian interpretation aligns naturally with our probabilistic framework, where the posterior distribution represents our updated belief about the parameters after observing the experimental FRF data.

The threshold value α=1.1 in Equation (32) was calibrated to accommodate the combined experimental and modelling uncertainties observed in our setup. Specifically, the pristine beam (Beam 1) was used to quantify discrepancies between the baseline numerical model and experimental measurements. The total uncertainty was estimated at 6–12%, originating from: (i) laser vibrometer measurement noise (∼2%), (ii) model simplifications in boundary conditions and damping (∼4–8%), and (iii) material property tolerances (∼2%). The value α=1.1 (i.e., a 10% allowance) was selected to conservatively bound this uncertainty range while maintaining physical plausibility. Although this specific value was calibrated for our experimental configuration, the calibration methodology is general: users should characterise their own uncertainty sources using a baseline structure and set α accordingly. For typical vibration-based SHM applications with moderate modelling fidelity, α values between 1.05 and 1.15 are often appropriate.

This phased and element-wise regularisation addresses two distinct requirements:

1. Mathematical Stability: The regularisation term R(ξ) prevents ill-posedness during identification.
2. Physical Plausibility: Post-convergence, element-level regularisation enforces that no stiffness value can exceed the undamaged baseline (with an uncertainty allowance), after which the statistical summary (mean and CI) is computed.

By separating these functions and applying the physical bounds to each identified EI value before final statistical aggregation, we avoid the algorithmic distortion that occurs when enforcing strict physical bounds during iterative sampling, while ensuring the final reported mean and CIs respect fundamental physical principles.

### 3.4. Optimal Sensor Placement for Informative Measurements

While the Bayesian framework with physical regularisation provides a robust mathematical foundation for damage identification, its practical efficacy depends critically on the quality of the experimental data. In a laboratory or real-world setting, it is infeasible to instrument every degree of freedom of a structure. The choice of sensor locations therefore becomes a paramount concern, as poor placement can fail to capture the essential dynamic features needed to resolve the inverse problem. A strategically placed, limited set of sensors must be capable of informing the model to distinguish between healthy and damaged states effectively.

This subsection addresses the practical challenge of transitioning from a well-posed numerical model to a feasible experimental setup. We establish a strategy for optimal sensor placement to ensure that the limited measurements obtained are highly informative of the structural dynamic characteristics, thereby enabling the regularised Bayesian identification framework to perform successfully.

**Figure 2:**
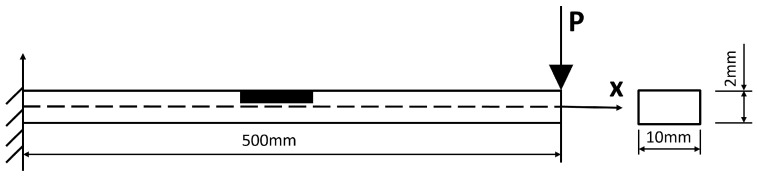
Degraded cantilever beam subjected to an external unit transverse pulse excitation with three sensors.

A cantilever beam (the black area denotes a potential SMD area) subject to a unit pulse excitation at its end of length L=0.5 m, width b=0.01 m, height h=0.002 m (Figure 2) is studied in this section. The cantilever beam is discretised into 100 elements, resulting in a total of 101 nodes. Each node has two degrees of freedom (DoFs): vertical translation and rotation, leading to a total of 202 DoFs.

Given the infeasibility of measuring all 202 DoFs in our discretised cantilever beam model, strategic sensor placement becomes crucial to maximise information content while minimising computational requirements.

**Figure 3:**
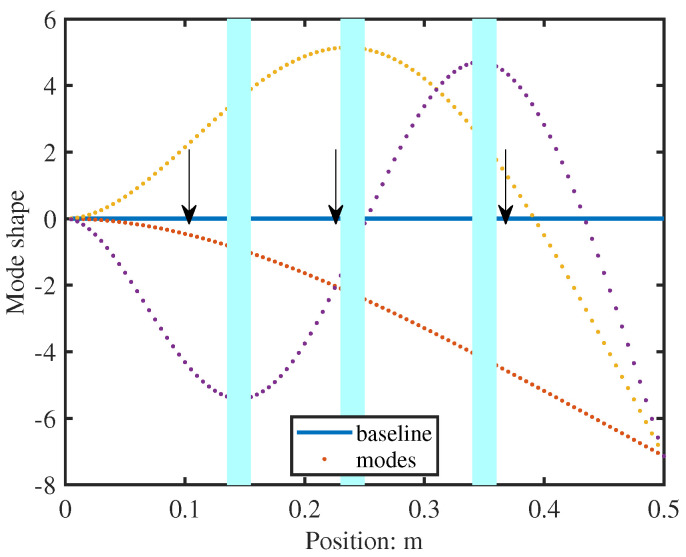
Suitable sensor positions indicated by the cyan areas.

**Figure 4:**
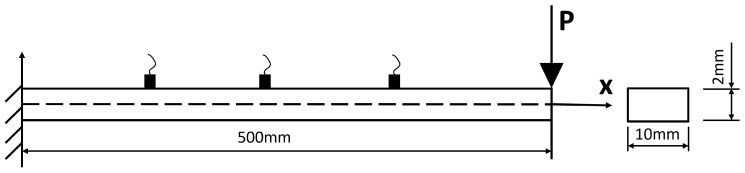
The recommended sensor positions for SMD identification.

Since low-frequency structural response is dominated by fundamental vibration modes, sensors must capture these mode shapes effectively. With the measured frequency range below 200 Hz, the first three modes govern the dynamic behaviour. To capture the essential dynamic characteristics, sensors should be positioned at anti-nodes (points of maximum displacement) of the dominant modes. Figure 3 illustrates the optimal sensor placement regions, with cyan areas highlighting locations corresponding to mode shape peaks. The recommended configuration, shown in Figure 4, positions sensors at approximately $\frac{L}{3}$, $\frac{L}{2}$, and $\frac{2L}{3}$ along the beam length to ensure comprehensive mode characterisation.

## 4. Experimental Methodology

This section outlines the experimental procedure for validating the Bayesian identification algorithm applied to a cantilever beam. The objectives were to: (1) calibrate a baseline numerical model using a pristine beam, and (2) select the optimal measurement apparatus for subsequent damage identification.

### 4.1. Experimental Setup and Specimens

**Figure 5:**
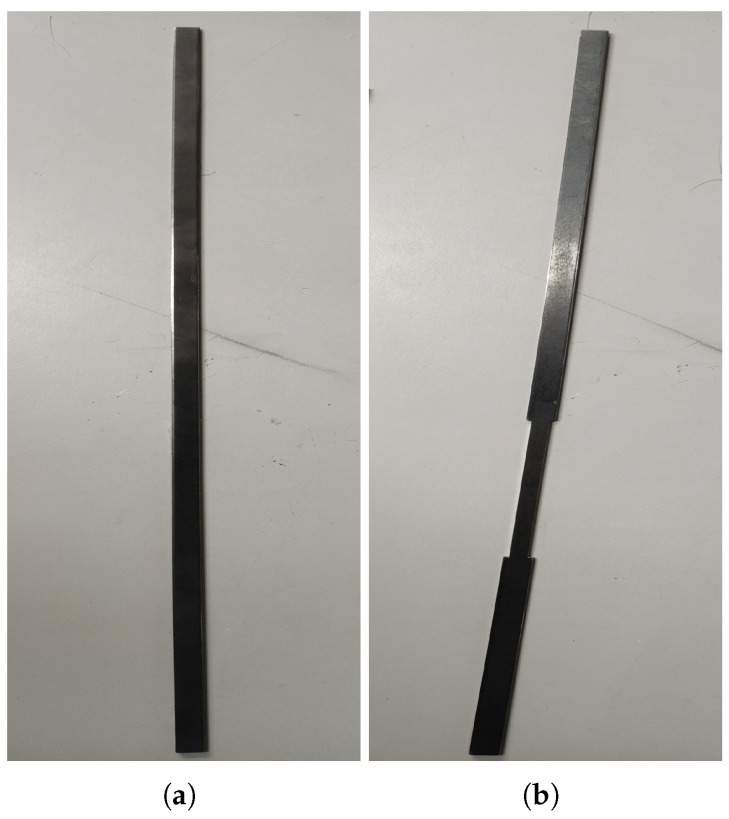
The experimental specimens: (**a**) The pristine beam (Beam 1). (**b**) The beam with a pair of symmetric open-edge cut-outs (Beam 2). Both beams have length L=0.5 m, width b=10 mm, and thickness h=2 mm. The damaged region in Beam 2 is located 0.1 m from the fixed end and extends 0.1 m along the span, with each cut-out being 2 mm wide and extending through the full thickness (see Figure 7 for detailed geometry).

**Figure 6:**
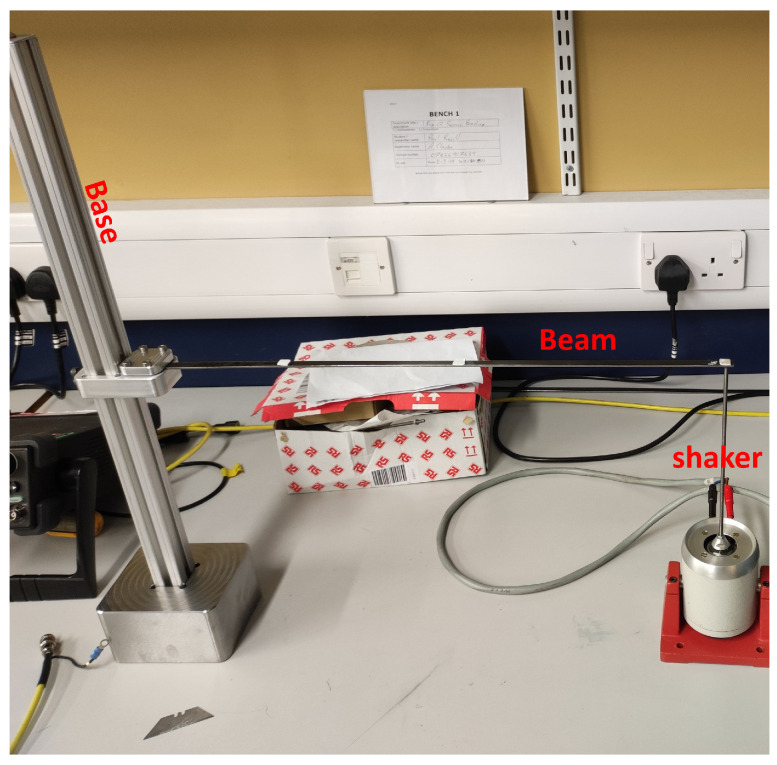
The experimental setup for a cantilever beam subjected to an exciting shaker at its free end.

**Figure 7:**
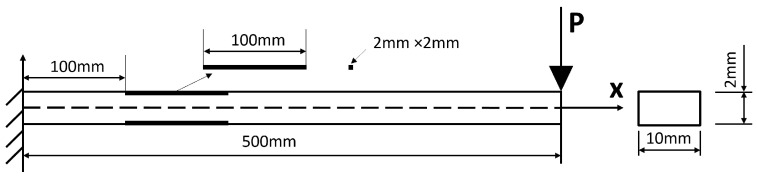
Schematic of the symmetric open-edge cut-out damage in Beam 2. The damaged region was located with its nearest edge 0.1 m from the fixed end. Each cut-out had length $\ell_{d}=100$ mm, width $w_{d}=2$ mm, and depth equal to the full thickness h=2 mm (i.e., complete through-thickness removal).

Two cantilever beam specimens were tested: Beam 1 (pristine) and Beam 2 (with two symmetrical open-edge cut-outs to simulate a single degraded region). Both beams were manufactured from black mild steel with nominal dimensions: length L=0.5 m, width b=10 mm, and thickness h=2 mm. The damage in Beam 2 consisted of two identical rectangular cut-outs machined symmetrically from each edge, creating a locally reduced cross-section. Each cut-out had length $\ell_{d}=100$ mm, width $w_{d}=2$ mm, and depth equal to the full thickness h=2 mm (i.e., complete through-thickness removal). The damaged region was located with its nearest edge 0.1 m from the fixed end, resulting in a central weakened zone spanning approximately x∈[0.1, 0.2] m. The intact web between the cut-outs was 6 mm wide, giving a total moment of inertia reduction of approximately 40% within the damaged region relative to the pristine section. These dimensions were selected to produce a measurable but non-catastrophic stiffness reduction representative of early-stage degradation. The specimens and the general test setup are shown in Figure 5 and Figure 6, respectively; a detailed schematic of the damage geometry is provided in Figure 7. The symmetric cut-outs reduce the second moment of area within the damaged zone from the pristine value $I_{0}=bh^{3}/12=6.67\times 10^{-12}\;m^{4}$ to $I_{d}=4.00\times 10^{-12}\;m^{4}$, corresponding to a 40% local reduction in bending stiffness (EI). For the entire cantilever beam (length L=0.5 m with damage over $L_{d}=0.1$ m), the overall stiffness reduction is approximately 8%, calculated via series combination of the damaged and pristine segments. This configuration provides a clear yet moderate stiffness loss signature, ideal for evaluating the inverse identification framework’s sensitivity to localised degradation.

**Figure 8:**
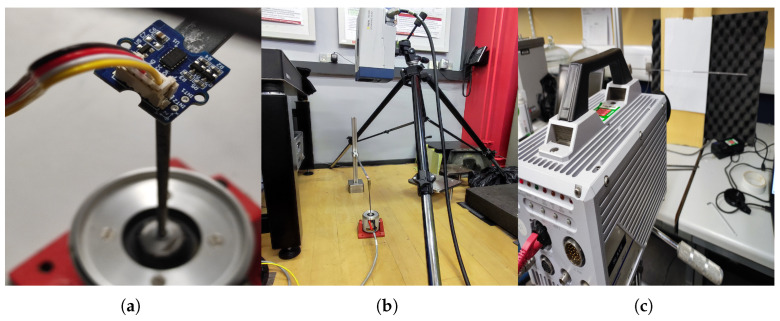
The experimental setup for the digital sensors: (**a**) A close-up of the 3-axis accelerometer. (**b**) The experimental setup of the laser vibrometer test. (**c**) The close-up of the high-speed camera.

Two cantilever beam specimens were tested: Beam 1 (pristine) and Beam 2 (with two symmetrical open-edge cut-outs to simulate one-degraded area). The specimens and the general test setup, which involved a shaker for free-end excitation, are shown in Figure 5 and Figure 6, respectively. To collect robust data for model calibration, three measurement techniques were evaluated: a 3-axis digital accelerometer (ADXL345), a PSV-500-3D scanning laser vibrometer, and a FASTCAM SA 1.1 high-speed camera (Figure 8).

### 4.2. Apparatus Comparison and Selection

Free vibration tests were conducted to determine the natural frequencies of both beams and to critically assess the three measurement apparatuses. A summary of their key characteristics and performance in this study is provided in Table 1.

**Figure 9:**
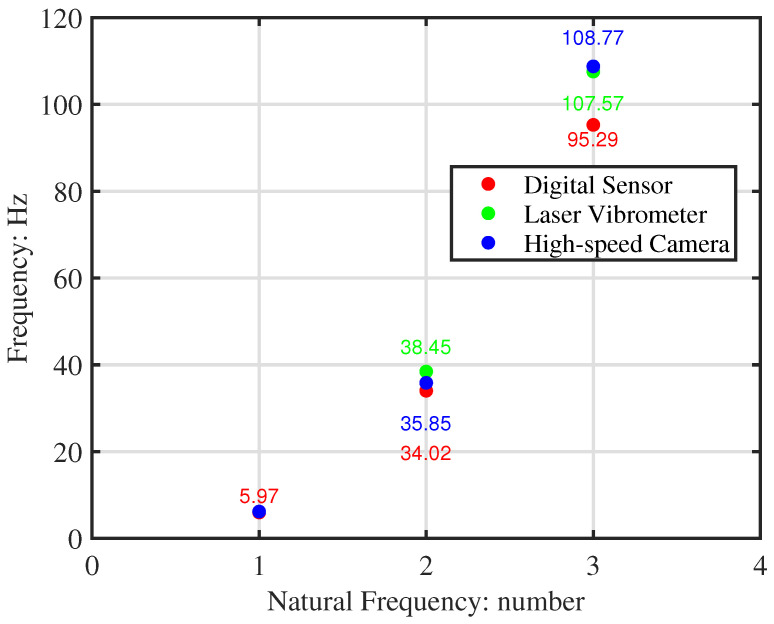
Comparison of natural frequencies for Beam 1 obtained from the three apparatuses, highlighting the mass-loading effect of the accelerometer.

The natural frequencies extracted from each apparatus are listed in Table 2. The accelerometer data shows a clear downward shift compared to the non-contact methods, a discrepancy attributed to sensor mass and cable damping. This is visually confirmed in Figure 9, which plots the natural frequencies of the pristine beam from all three tests.

Based on this comparative analysis in Table 1, the laser vibrometer was selected for this study. Its non-contact nature eliminated mass-loading errors, its high sampling rate ensured data fidelity, and its operational practicality allowed for the extended monitoring periods required for forced vibration tests.

### 4.3. Initial Model Calibration Based on Natural Frequencies

The baseline finite element model was calibrated using the laser vibrometer data from the pristine Beam 1. The beam was manufactured from black mild steel, with material property ranges provided in Table 3. The model was updated to match the experimental natural frequencies. The resulting calibrated Young’s modulus of $E=1.99\times 10^{11}\;\mathrm{Pa}$ falls within the expected range for black mild steel. The calibrated density of $\rho =8045.05\;{\mathrm{kg}/m}^{3}$ is slightly higher than the typical range. This minor deviation is a recognised phenomenon in model updating, where parameters can adjust beyond nominal bounds to compensate for simplifications in the model, such as ideal boundary conditions or unaccounted mass, to accurately replicate the experimental dynamics.

### 4.4. Forced Vibration Testing and FRF Calculation Methodology

This section details the experimental and numerical procedures for characterising the dynamic behaviour of the cantilever beams. Following the initial model calibration based on natural frequencies (Section 4.3), forced vibration tests were conducted to obtain Frequency Response Functions (FRFs). These experimental FRFs were then used to further calibrate and refine the finite element model, addressing aspects such as damping characteristics and boundary condition effects that are not fully captured by natural frequency data alone. The refined model, validated against both natural frequencies and FRF data, subsequently served as the reliable baseline for damage identification.

The cantilever beam was excited at its free end using a sweeping sinusoidal signal p(t)=sin(2πft), where the frequency *f* ranged from 1 Hz to 100 Hz. We accounted for the shaker’s support stiffness using the Lagrange Multiplier Method to accurately simulate the experimental boundary conditions.

**Figure 10:**
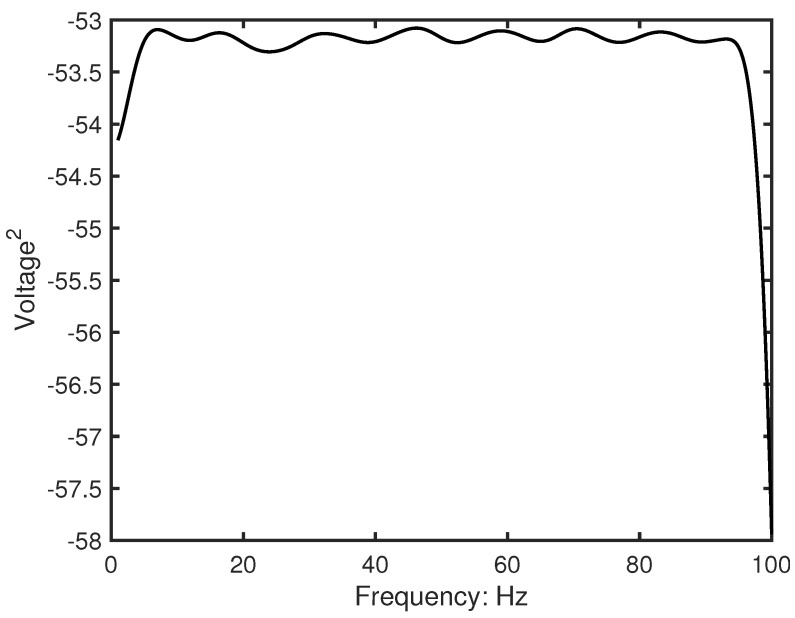
Auto-power spectral density of the sweep excitation signal, demonstrating near-constant power distribution across the frequency range of interest (1–100 Hz). This characteristic validates the experimental FRF processing methodology, where the velocity response power spectrum can be directly related to FRF amplitude under constant-power excitation conditions.

Under sweep excitation conditions, the auto-power spectral density of the excitation signal was approximately constant in the frequency domain (Figure 10), i.e., $S_{ff}\left(\omega \right)\approx \mathrm{Constant}$. This characteristic establishes a clear proportional relationship between the velocity response power spectrum and the FRF amplitude:

(33) $$S_{vv}\left(\omega \right)={\left|H_{M}\left(\omega \right)\right|}^{2}\times S_{ff}$$

Therefore,

(34) $$|H_{M}\left(\omega \right)|\propto \sqrt{S_{vv}\left(\omega \right)}$$

Based on this relationship, the experimental data processing uses the square root of the velocity auto-power spectrum as an equivalent measure of FRF amplitude:

(35) $${\mathrm{FRF}}_{\mathrm{amplitude}}=\sqrt{S_{vv}\left(\omega \right)}$$

where $S_{vv}\left(\omega \right)$ is calculated using Welch’s method.

This method effectively avoids the difficulties associated with traditional FRF calculations that require precise force sensor data, while ensuring reliable results under sweep excitation conditions.

The numerical model directly calculates the velocity/force FRF (mobility) following the logic of Equation (21).

Considering the constant power characteristics of sweep excitation, simulation and experimental outputs can be directly compared through normalisation processing. The comparison between experimental and numerical FRFs was performed by scaling the numerical FRF to match the experimental amplitude reference. The scaling factor *a* was determined from the ratio of maximum amplitudes:

(36) $$a=\frac{\max|\sqrt{S_{vv}\left(\omega \right)}|}{\max|H(\omega )|},$$

The scaled numerical FRF for comparison is then:

(37) $$H_{\mathrm{sim},\mathrm{scaled}}\left(\omega \right)=a\cdot \left|H\left(\omega \right)\right|,$$

while the experimental FRF maintains its original amplitude scale:

(38) $$H_{\exp}\left(\omega \right)=\sqrt{S_{vv}\left(\omega \right)}.$$

This scaling approach preserves the physical amplitude relationships in the experimental data while enabling direct quantitative comparison with numerical predictions. The method ensures consistent evaluation of resonance frequencies, mode shapes, anti-resonance locations, and relative amplitude distributions across the frequency range of interest.

**Figure 11:**
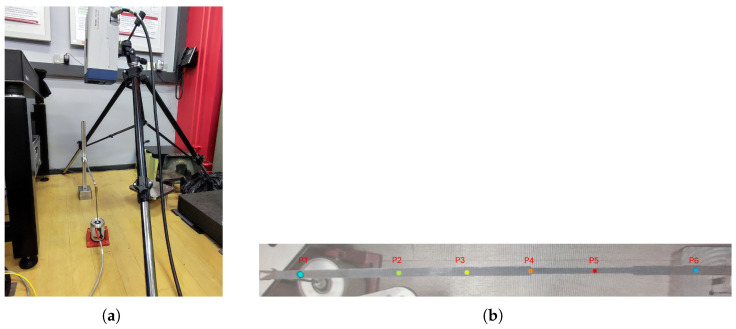
Laser vibrometer experimental setup and measurement positions. (**a**) Experimental setup; (**b**) Measurement positions P1–P6.

**Figure 12:**
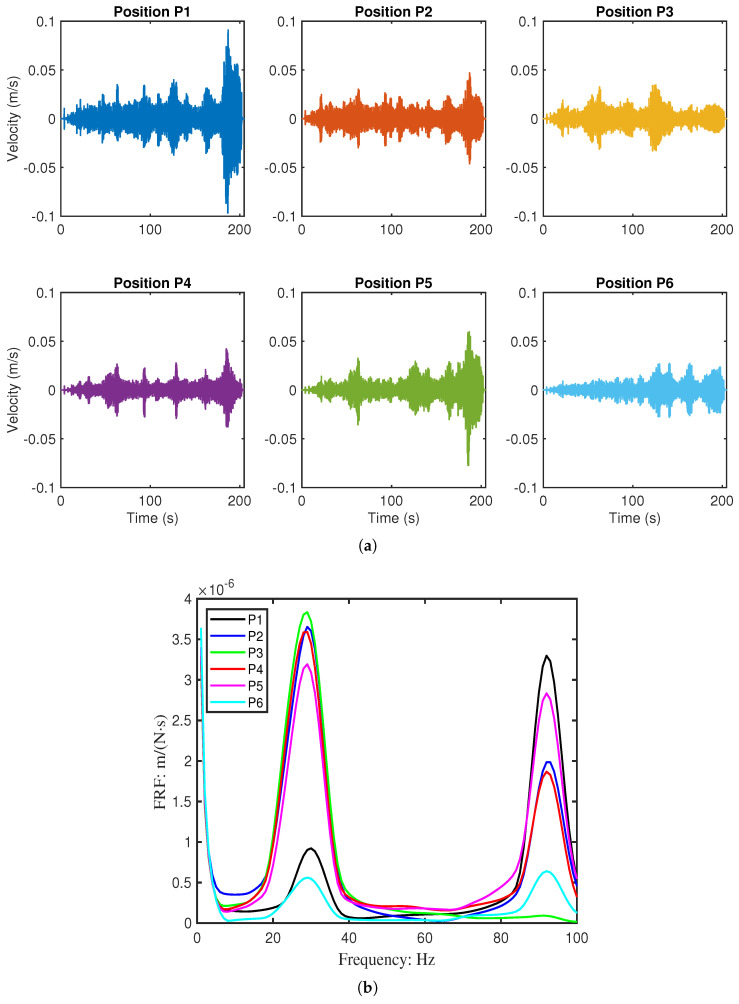
Measured the dynamic response of Beam 1 (pristine) at six discrete points (P1–P6). (**a**) Time-domain velocity signals under swept-sine excitation. (**b**) Corresponding mobility Frequency Response Functions (FRFs).

**Figure 13:**
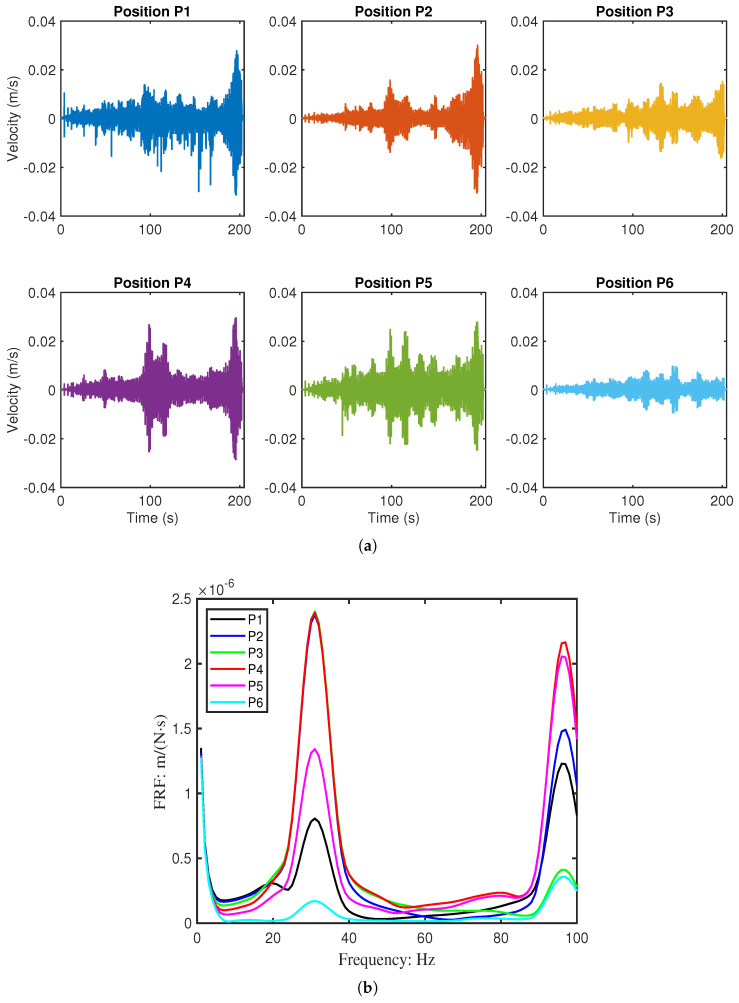
Measured the dynamic response of Beam 2 (pristine) at six discrete points (P1–P6). (**a**) Time-domain velocity signals under swept-sine excitation. (**b**) Corresponding mobility Frequency Response Functions (FRFs).

Vibrations were measured at six points (P1–P6) along the beam using a laser vibrometer, as shown in Figure 11. The resulting velocity responses and corresponding mobility FRFs are presented in Figure 12 and Figure 13. Table 4 summarises the first two resonant frequencies, while Table 5 provides the damping coefficients. Notably, Point 6 near the fixed end showed significantly lower energy, and Point 3 exhibited reduced energy at the second mode, consistent with expected mode shape behaviour.

### 4.5. Model Refinement and Validation

The pristine FE model was refined to reflect the experimental boundary and excitation conditions. The model refinement proceeded through an iterative process comparing experimental and numerical FRFs. Key parameters adjusted during refinement included:

1. Damping coefficients: Rayleigh damping parameters were tuned to match the amplitude and bandwidth of resonance peaks observed in experimental FRFs.
2. Boundary conditions: The stiffness of the shaker support was refined using the Lagrange Multiplier Method to better match experimental boundary conditions.
3. Local stiffness variations: Minor adjustments were made to account for localised effects observed near the excitation point.

**Figure 14:**
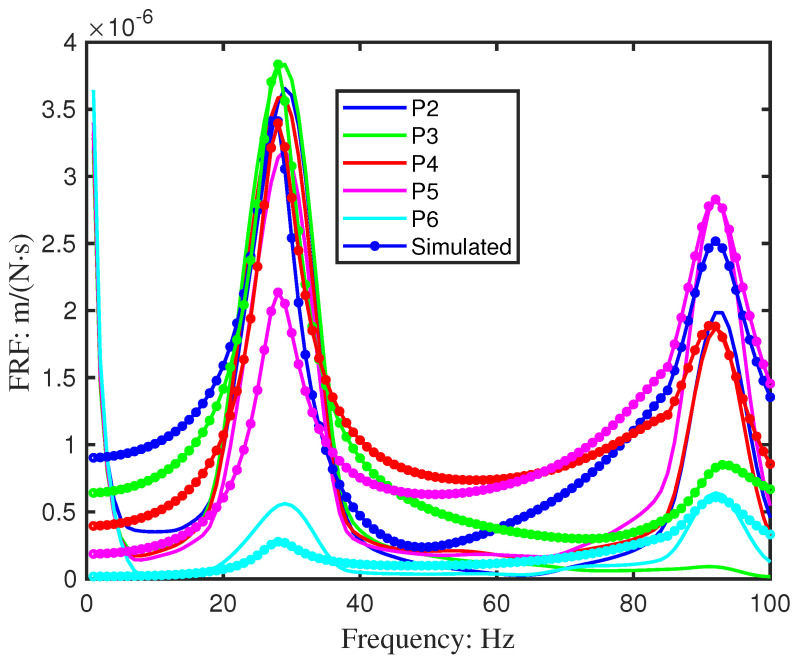
The mobility FRF of experimental and simulated velocities for beam 1 (Point 1 excluded).

Damping significantly affects FRF amplitudes, especially near resonances. To align simulated and experimental FRFs for Beam 1, a frequency-dependent damping adjustment was employed. Rayleigh damping parameters were tuned specifically around the first two resonant frequencies where velocity-dependent damping effects are most pronounced. This targeted calibration—rather than applying a uniform damping model across the entire spectrum—yielded good agreement in both amplitude and peak shape for the pristine beam (Figure 14). The success of this calibration supports the assumption that the damping mechanism remains largely unchanged for the geometric damage introduced in Beam 2, validating the use of the same damping model for the subsequent inverse identification.

The refinement was considered complete when the normalised FRFs demonstrated consistent agreement in resonance frequencies, mode shapes, and anti-resonance locations, as quantified by correlation coefficients exceeding 0.95 for the frequency range of interest.

Experimental FRFs exhibited flattened resonance peaks, indicating velocity-dependent damping behaviour. Estimated damping ratios showed that the first mode exhibited approximately three times the damping of the second mode (Table 5). In addition, the excitation signal displayed a near-constant auto-power density across the frequency range (Figure 10), confirming that the input amplitude was effectively independent of frequency; therefore, the measured velocity responses were directly applicable for numerical calibration.

**Figure 15:**
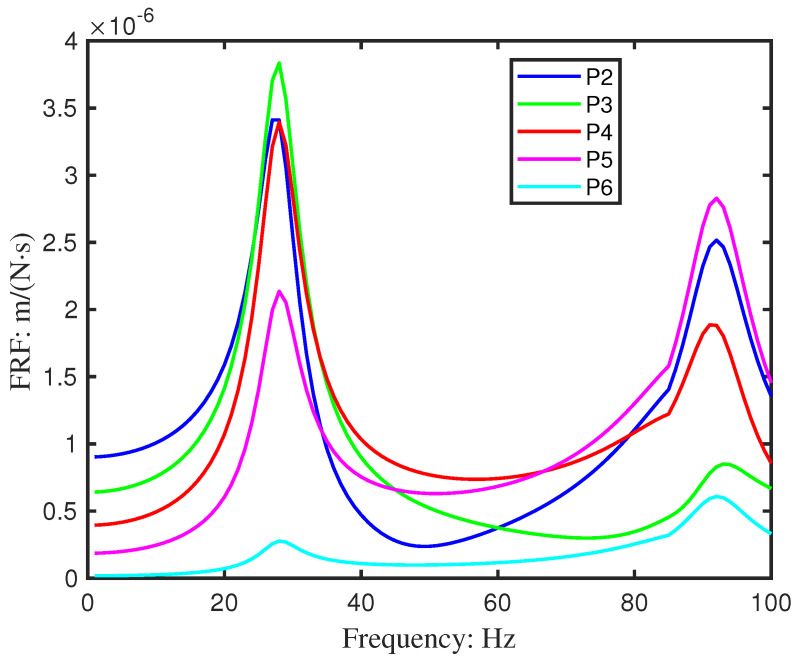
The velocity FRF from the numerical model for the pristine beam (Point 1 excluded).

Simulated auto-power spectra reproduced the general dynamic characteristics observed experimentally (Figure 15). However, results obtained at Point 1, located adjacent to the shaker, showed notable deviation, likely due to local interaction effects. This point was therefore excluded, yielding close agreement across the remaining sensor locations (Figure 14).

**Figure 16:**
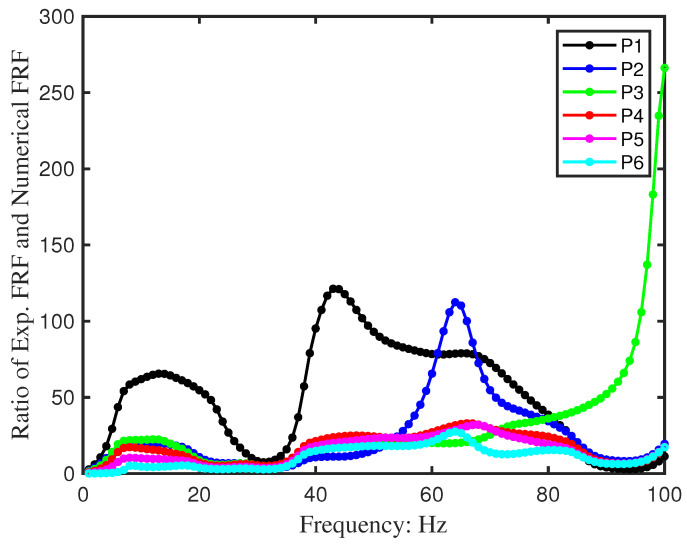
The calibration curve of the numerical model. This curve is used to calibrate the numerical FRF to align with the experimental FRF, thus helping to regularise the inverse identification.

To further improve prediction quality, a calibration curve was constructed from the ratio of experimental to simulated responses (Figure 16). The calibration curve can be applied to adjust the numerical FRF during the iteration for the SMD identification for Beam 2, which would help to overcome the simulation imperfection.

## 5. SMD Identification Results and Discussion

This section presents the application of the proposed Bayesian inverse identification framework—with the two-phase constraint strategy outlined in Section 3.2 and Section 3.3—to experimental FRF data from Beam 2, which contains a single symmetrical open-edge cut-out to simulate localised SMD. The objectives are to: (i) validate the convergence and uncertainty quantification of the identified parameters, (ii) evaluate the effectiveness of the constraint strategy in stabilising the ill-posed inverse problem, and (iii) demonstrate accurate damage localisation while ensuring physical plausibility.

**Figure 17:**
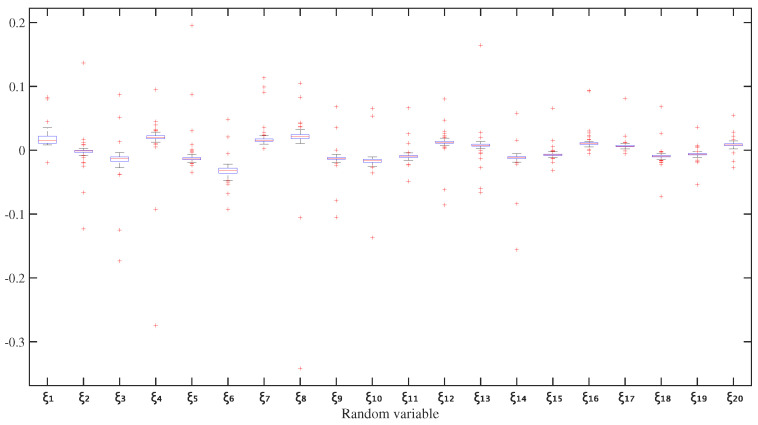
Statistical distribution of identified random variables ξ from 100 iterations. The boxplots summarise the median, quartiles, and outliers for each of the twenty random variables used in the spectral model-driven identification.

**Figure 18:**
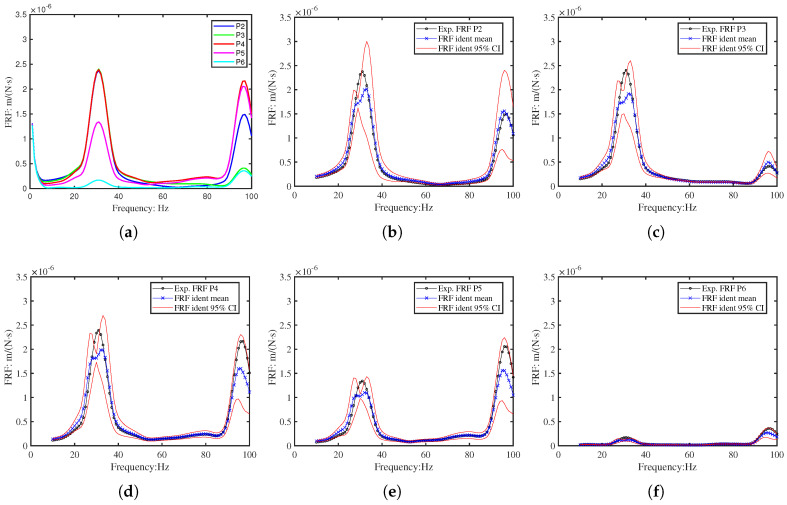
The experimental FRF of P2-P6 for beam 2 and the corresponding identified FRF: (**a**) The experimental M-FRF of beam 2 at points P2–P6; (**b**–**f**) The identified FRF 95% CI for beam 2 at points P2–P6.

For the inverse identification, twenty random variables (ξ) were employed. Figure 17 presents the statistical distribution of these variables over 100 identification iterations. The results, detailed in Figure 18, demonstrate that the identified FRFs converge towards the target experimental FRFs at each measurement position (P2–P6). Notably, the 95% credible interval of the identified FRFs encompasses the target data at all points, confirming the method’s robustness despite the peak splitting observed in the numerical model.

### 5.1. Two-Phase Constraint Implementation and Outcomes

**Figure 19:**
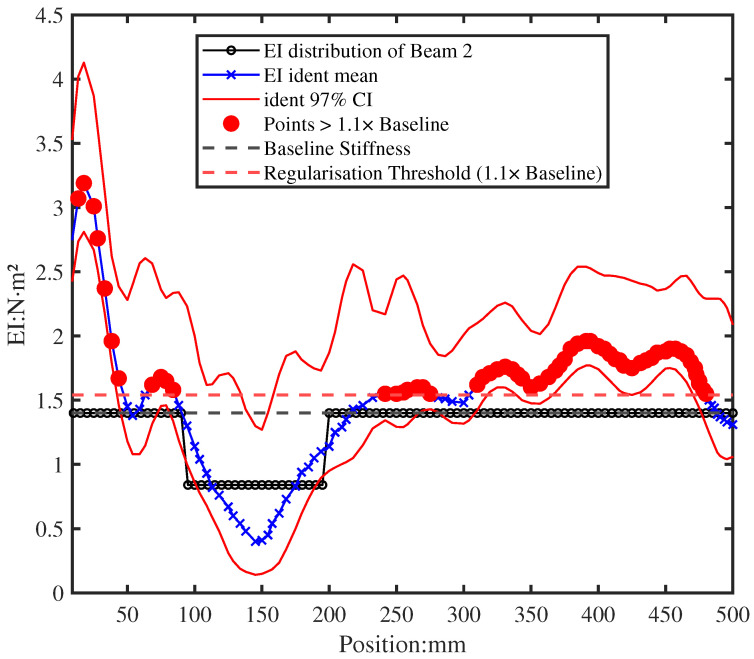
Initial EI identification with moderate regularisation yields stable but physically imperfect results, including non-physical stiffness enhancements outside the damage region.

Figure 19 utilised a moderate regularisation during the identification iteration that assumed a global stiffness reduction, with the total identified rigidity constrained such that $\sum \kappa_{M}=0.9\sum \kappa_{\mathrm{base}}$. This 10% reduction factor was selected based on preliminary experimental observations and engineering judgment, reflecting typical stiffness loss magnitudes in early-stage damage scenarios without over-constraining the solution space. This average of the 100 identified EI successfully located the damage region at approximately X∈[0.1, 0.2]m. The identified stiffness reduction within the damage region (approximately 35–45% from Figure 19) aligns well with the actual 40% local reduction, demonstrating the method’s accuracy in quantifying damage severity despite the modest 8% global stiffness change. However, it also produced physically impossible local stiffness enhancements in other regions (marked by the red dot), with pointwise values of $\kappa_{S}$ reaching up to $4.2\;{N\cdot m}^{2}$.

A noteworthy observation is the significant overestimation of EI at the fixed end compared to the baseline. This systematic phenomenon, consistently observed across identifications, suggests either imperfect boundary condition simulation or parameter insensitivity in this region, where bending rigidity variations minimally affect the measured and numerical FRFs. Despite this boundary artefact, the damage region remains clearly identifiable, demonstrating the method’s robustness for damage localisation away from boundary-influenced zones.

The presence of such boundary artefacts motivated the application of the post-convergence physical bound enforcement described in Section 3.3, which selectively suppresses these non-physical enhancements while preserving the identified damage signature.

**Figure 20:**
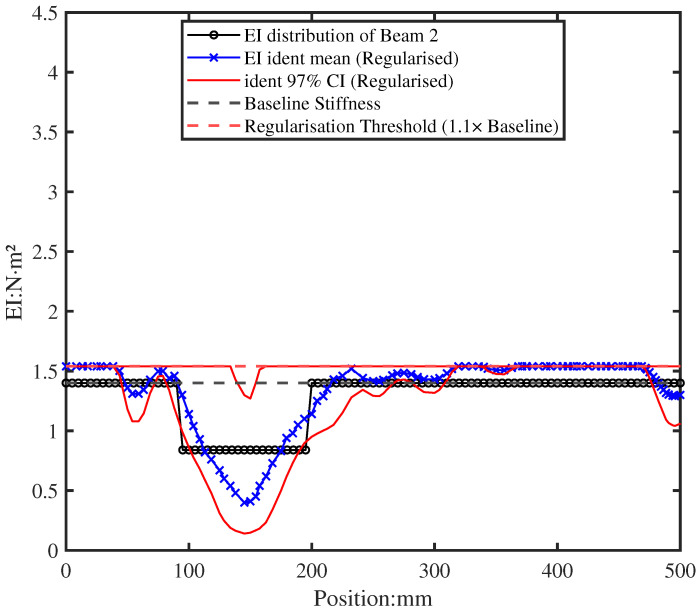
Final identified EI distribution after post-convergence regularisation with α=1.1 (calibrated to accommodate 6–12% combined uncertainty). The mean EI profile (blue line) clearly indicates the damage location and severity, while physical violations have been eliminated.

To address the non-physical enhancements from the moderate regularisation, the post-convergence regularisation rule (Equation (32)) was applied individually to each identified EI sample. Figure 20 presents the final, physically plausible EI distribution after regularisation. The mean EI profile clearly reveals the location and extent of the simulated damage, while spurious stiffness enhancements have been suppressed. This two-phase approach—combining moderate regularisation during identification with targeted post-processing—maintains accurate damage localisation while enforcing the physical principle that degradation reduces, rather than enhances, structural stiffness.

### 5.2. Post-Regularisation FRF Evaluation

To complete the validation loop, FRFs were recalculated from the regularised EI fields. For each regularised stiffness sample $\kappa_{\mathrm{reg}}^{\left(i\right)}\left(x\right)$, the corresponding FRF is obtained by solving the deterministic forward problem:

$$Z\left(\omega ,\kappa_{\mathrm{reg}}^{\left(i\right)}\right)\;U\left(\omega \right)=F\left(\omega \right),\;H_{\mathrm{post}}^{\left(i\right)}\left(\omega \right)=R\;U\left(\omega \right),$$

where Z is the dynamic stiffness matrix constructed from $\kappa_{\mathrm{reg}}^{\left(i\right)}\left(x\right)$, U is the displacement vector, F is the force vector, and R selects sensor locations. This step does not involve the KL coefficients ξ; it uses only the physically regularised EI field $\kappa_{\mathrm{reg}}\left(x\right)$ as input to the forward solver.

**Figure 21:**
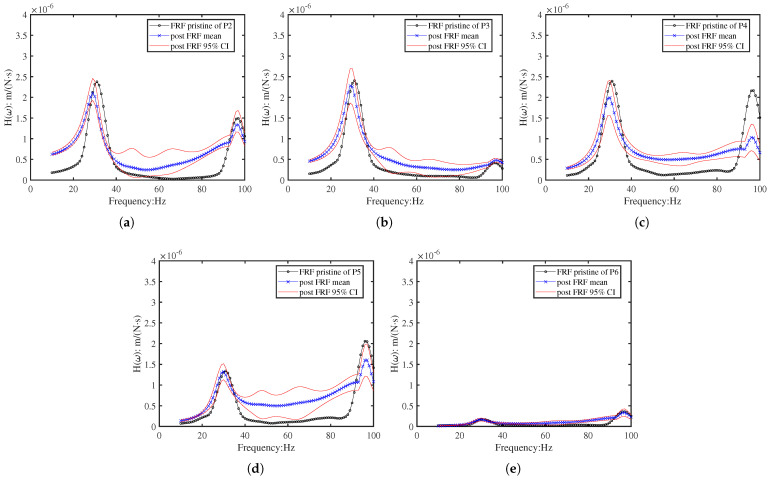
Post-regularisation FRFs recalculated from the physically constrained EI samples. Although the amplitude matching is reduced due to the enforced physical bounds, the resonant peak locations remain aligned, confirming preservation of essential damage signatures. (**a**) Position P2; (**b**) Position P3; (**c**) Position P4; (**d**) Position P5; (**e**) Position P6.

The results are shown in Figure 21. The mismatch between the post-regularisation FRFs and the experimental data illustrates a fundamental insight: overly stringent physical constraints would yield distorted identification results. This discrepancy arises because no numerical model can perfectly replicate real-world conditions, and unaccounted measurement and modelling uncertainties inevitably exist.

Nevertheless, the preserved alignment of resonant peaks across all sensor positions confirms the feasibility of the inverse identification framework. This spectral coherence indicates that despite the inevitable imperfections in model fidelity, the essential dynamic signature of structural damage remains extractable. This is precisely why the two-phase approach succeeds: it avoids imposing rigid physical constraints during the identification process (which would prevent convergence), while still ensuring the final stiffness field respects physical principles after convergence.

Thus, the observed FRF mismatch does not undermine the method’s validity; rather, it validates the core design rationale. The two-phase strategy acknowledges the inherent limitations of model-based identification while delivering physically plausible damage localisation—the ultimate goal of this work.

This work introduces a two-phase constraint strategy that decouples mathematical stabilisation during Bayesian inference from physical plausibility enforcement after convergence. Unlike traditional single-phase regularisation—which often forces a trade-off between algorithmic stability and physical realism—our approach applies moderate regularisation during Hamiltonian Monte Carlo sampling to ensure well-posedness, then enforces element-wise physical bounds (e.g., prohibiting stiffness enhancements) only after the posterior has been sampled. This separation prevents convergence distortion while guaranteeing that final stiffness estimates respect the fundamental principle that damage reduces, rather than increases, structural rigidity. The calibrated threshold α=1.1 further accommodates experimental uncertainties (6–12%), offering a balanced solution to a long-standing challenge in FRF-based damage identification.

## 6. Conclusions

This study presents a novel vibration-based probabilistic damage identification approach which can capture any profile or shape of damage at multiple sites based on its vibration response. The approach does not rely on heavily informative priors for structural material degradation (SMD) identification. Instead, it demonstrates for the first time the applicability and performance accuracy a random field model for inverse identification of any arbitrary distribution of SMD along the span of a structure using only a few stochastic parameters. By modelling SMD as a low-dimensional stochastic process, the method achieves significant computational savings, which reduce parameter dimensionality by orders of magnitude, while maintaining high spatial resolution. By reconstructing the full degradation field from a compact set of dominant KL parameters, the framework enables efficient identification of damage, whether it is localised (e.g., cracks, notches) or distributed (e.g., corrosion, uniform wear). The approach has been verified with experimental studies. It shows that tapping the vibration measurement data at only a handful of points is sufficient for an accurate identification of SMD.

This study presents a novel stochastic framework for structural material degradation (SMD) identification that efficiently couples Karhunen–Loéve (KL) expansion with Frequency Response Function (FRF) analysis. By modelling SMD as a low-dimensional stochastic process, the method achieves significant computational savings, which reduce parameter dimensionality by orders of magnitude, while maintaining high spatial resolution. By reconstructing the full degradation field from a compact set of dominant KL parameters, the framework enables efficient identification of damage, whether it is localised (e.g., cracks, notches) or distributed (e.g., corrosion, uniform wear).

A key methodological contribution is the development of a two-phase constraint strategy that resolves the inherent tension in inverse problems: the need for mathematical stabilisation versus the risk of physical over-constraint. The phased approach separates:

Phase 1—Mathematical Stabilisation: Moderate regularisation during Hamiltonian Monte Carlo sampling stabilises the ill-posed problem without distorting convergence.
Phase 2—Physical Enforcement: Post-convergence selective suppression removes non-physical stiffness enhancements, enforcing the principle that damage reduces—not increases—structural rigidity.

This separation proved essential, with the calibrated α=1.1 threshold accommodating experimental uncertainties of 6–12% while preserving damage signatures.

Experimental validation on cantilever beams demonstrated successful localisation and quantification of a single damage region using laser vibrometry-derived FRFs. The method provided both statistically credible intervals (95%) and physical plausibility, addressing a critical limitation of traditional SHM approaches that often yield mathematically valid but physically impossible results.

Two primary challenges were identified for practical deployment: (1) the critical influence of damping modelling accuracy on FRF-based identification, and (2) the increased complexity of identifying multiple concurrent damage regions. Future work will extend the framework to multi-damage scenarios, refine damping representations, and test robustness under varied boundary conditions and noise levels.

The proposed framework represents a significant step toward reliable, physics-informed structural health monitoring, offering a balanced approach to uncertainty quantification and physical constraint enforcement that enhances both mathematical rigour and practical utility in real-world applications. Future work will extend the framework to multi-damage scenarios, refine damping representations, and test robustness under varied boundary conditions and noise levels, contributing to the ongoing development of robust numerical methods for inverse problems in engineering [28].

### Limitations and Outlook for Practical Application

The experimental validation presented in this study was conducted on a laboratory-scale cantilever beam with a single, well-defined damage region. This controlled setting was essential for validating the core components of the proposed stochastic identification framework—the KL-based parameterisation and the two-phase constraint strategy—under known conditions. However, the transition to practical, large-scale structural health monitoring (SHM) applications presents several challenges that must be acknowledged and addressed in future work.

1. Boundary Condition Uncertainty: Real-world structures rarely possess idealised boundary conditions. Semi-rigid connections, foundation flexibility, and interaction with adjacent components introduce significant uncertainty. While the current model assumed a fixed base, the framework is generalisable. Future extensions could parameterise boundary stiffnesses within the vector ξ for joint identification, or apply the KL expansion to represent spatial uncertainty in support properties.
2. Computational Scalability: For large-scale structures, the computational cost of repeated forward solves (FRF calculations) within the HMC loop becomes the primary bottleneck, not the parameter dimensionality (which is controlled by the KL truncation). This can be mitigated by integrating model reduction techniques (e.g., modal truncation, Krylov subspace methods) for the forward model, and by exploiting the inherent parallelism of Monte Carlo sampling.
3. Complex Geometry and Incomplete Measurements: The optimal sensor placement strategy in Section 3.4 is a first step towards dealing with limited measurements. For complex 2D or 3D structures, advanced placement algorithms (e.g., based on effective independence or information entropy) will be necessary to maximise the information content of sparse sensor data. The KL expansion itself is directly applicable to random fields on complex domains, provided an appropriate covariance kernel is defined.
4. Interpretation in Multi-Damage Scenarios: Identifying multiple, interacting damage sites increases the ill-posedness of the inverse problem. The Bayesian approach provides a natural advantage here by quantifying uncertainty through the full posterior distribution. The outcome is not a single deterministic damage map, but a probabilistic diagnosis—a set of plausible damage scenarios with associated credibility. This is precisely the form of information required for risk-based maintenance decisions in practical SHM.

In summary, while the current implementation demonstrates the method’s efficacy on a canonical problem, its architectural components—stochastic parameterisation, Bayesian updating, and phased constraint enforcement—are designed with scalability in mind. Future work will focus on integration with reduced-order modelling, validation on more complex structural systems (e.g., plates, frames), and the development of automated, interpretable post-processing tools for high-dimensional posterior distributions.

## Figures and Tables

**Table 1:** Comparison of measurement apparatuses.

Apparatus	Measured Quantity	Sampling Rate	Key Findings
Digital Accelerometer	Acceleration	500 Hz	Significant mass-loading effect, indicated by consistently lower natural frequencies (Figure 9).
Laser Vibrometer	Velocity	1024 Hz	Non-contact; high-fidelity data; minimal noise; suitable for long-duration tests.
High-Speed Camera	Displacement	1000 Hz	Non-contact, but limited by recording duration and stringent lighting requirements.

**Table 2:** Measured natural frequencies (Hz) from free vibration tests.

Beam	Apparatus	$f_{1}$	$f_{2}$	$f_{3}$
Beam 1 (Pristine)	Accelerometer	5.97	34.02	95.29
	Laser Vibrometer	6.18	38.45	107.57
	High-Speed Camera	6.18	35.85	108.77
Beam 2 (Damaged)	Accelerometer	5.97	36.01	98.08
	Laser Vibrometer	6.18	41.24	111.95
	High-Speed Camera	5.98	40.44	100.00

**Table 3:** Material properties of black mild steel.

	Young’s Modulus (GPa)	Density (kg/m^3^)
Typical Range	190–210	7850–8000
Calibrated Value	199	8045

**Table 4:** The first two resonant frequencies (Hz) measured at six discrete positions along the beams and the average value from the laser vibrometer test.

Beam No.	Position 1	Position 2	Position 3	Position 4	Position 5	Position 6	Mean ±σ
1	29.84	29.24	28.75	28.55	28.85	29.05	29.05 ± 0.46
	92.17	92.47	91.08	92.17	92.07	92.07	92.01 ± 0.48
2	31.01	30.90	30.90	30.90	30.90	30.90	30.91 ± 0.04
	96.47	96.69	96.47	96.58	96.47	96.47	96.53 ± 0.09

**Table 5:** Damping coefficients at different resonant frequencies for Beam 1 and Beam 2.

Beam No.	Position 1	Position 2	Position 3	Position 4	Position 5	Position 6	Mean ±σ
1	0.11	0.14	0.15	0.14	0.13	0.13	0.13 ± 0.01
	0.04	0.04	0.07	0.04	0.04	0.04	0.04 ± 0.02
2	0.11	0.11	0.11	0.11	0.11	0.11	0.11 ± 0.00
	0.03	0.03	0.03	0.03	0.04	0.03	0.03 ± 0.00

## Data Availability

Data can be shared when required.

## Abbreviations

The following abbreviations are used in this manuscript:

SMD	Structural Material Degradation
FRF	Frequency Response Function
KL	Karhunen–Loéve
SHM	Structural Health Monitoring
DS	Digital Sensor
LV	Laser Vibrometer
HC	High-speed Camera

## Appendix A. Hamiltonian Monte Carlo Implementation Details

The HMC sampling was implemented using MATLAB (R2023b)’s built-in hmcSampler function with the No-U-Turn Sampler (NUTS) variant. The complete implementation details, extracted directly from the sampler output, are summarised in Table A1 and discussed below.

**Table A1:** Complete HMC sampler configuration and diagnostics.

Parameter	Value
**Sampler Configuration**	
Sampler type	No-U-Turn Sampler (NUTS)
Step size (ϵ)	1.0925
Number of steps (*L*)	1 (NUTS dynamic determination)
Mass vector dimensions	21 × 1 (diagonal)
Mass vector range	[1.1685, 3.3558]
Mass vector mean	1.7160
Jitter method	jitter-both
Step size tuning	dual-averaging
Mass matrix tuning	iterative-sampling
Numerical gradient	Enabled
**Sampling Parameters**	
Number of parameters	21 (20$\xi_{i}$ + 1$\log\sigma^{2}$)
Burn-in iterations	500
Sampling iterations	3000
Total iterations	3500
Number of independent chains	100
Total posterior samples	63,000
**Convergence Diagnostics**	
Acceptance rate	0.67%
Effective sample size (min)	16.7
Effective sample size (mean)	17.4
Effective sample size (max)	17.7
Log-posterior mean	−0.2815
Log-posterior standard deviation	0.5387

The HMC sampler employed the No-U-Turn Sampler (NUTS) variant, indicated by L=1 leapfrog step, which dynamically determines trajectory length to avoid U-turns. The step size ϵ=1.0925 was automatically tuned during warm-up using the dual-averaging algorithm targeting a nominal acceptance rate of 65%. A diagonal mass matrix was adaptively tuned via iterative-sampling, with elements ranging from 1.1685 to 3.3558 (mean = 1.7160), reflecting the varying scales of the 21 parameters (20 Karhunen–Loève coefficients $\xi_{i}$ and 1 noise variance parameter $\log\sigma^{2}$). Numerical gradients were computed via finite differences to accommodate the non-differentiable components of the log-posterior.

A total of 100 independent HMC chains were executed from randomly initialised starting points $\xi^{\left(0\right)}∼N\left(0,0.1I_{21}\right)$. Each chain underwent 500 burn-in iterations for adaptation followed by 3000 sampling iterations, yielding 63,000 total posterior samples. The independent chains enabled robust assessment of convergence while providing comprehensive uncertainty quantification.

Convergence was rigorously evaluated using multiple diagnostics:

1 Acceptance rate: 0.67% across all samples. While lower than the typical optimal range of 60–80% for standard HMC, this reflects the challenging posterior geometry of the ill-posed inverse problem. The NUTS algorithm compensates through dynamic trajectory adjustment.
2 Effective sample size (ESS): Ranged from 16.7 to 17.7 independent samples per parameter, sufficient for reliable inference given the large total sample count (63,000).
3 Parameter stability: All 21 parameters showed stable posterior distributions with coefficients of variation <5% across chains (Table A1).
4 Trace inspection: Visual examination revealed proper mixing without divergent transitions.
5 Log-posterior convergence: Final log-posterior values stabilised at −0.28±0.54 after burn-in.

The sampling was parallelised across the 100 independent chains, with each chain requiring approximately 45 min on an Intel Xeon E5-2680 v4 processor (2.4 GHz, 14 cores). The total computational effort was approximately 75 CPU-hours.
